# On Some of the More Important Chemical Disinfectants

**Published:** 1853-03

**Authors:** George Wilson

**Affiliations:** Hon. Member of the Pharmaceutical Society of Great Britain


### SELECTIONS.

From the Pharmaceutical Journal (Eng.)

### *On some of the more Important Chemical Disinfectants.* By George Wilson, M.D., F.R.S.E., Hon. Member of the Pharmaceutical Society of Great Britain.

(*Concluded from last No.*)

3. *Chlorine.*—Of chlorine, which is at present the favorite disinfectant, it is needless to speak. Its peculiar power of decomposing combinations of hydrogen gives it, in one respect, a superiority over nitric acid, which does not decompose many of the gaseous hydro-carbons; but it should not be forgotten that it is only in the presence of light that this action of chlorine is fully displayed, so that its disinfectant influence is comparatively small in the case of dark or ill-lighted apartments, such as underground cellars, the lower cabins, or the hold of a ship, which are the very places where disinfectants are often most wanted.

4. *Aqua Regia*, as uniting the properties of nitric acid and of chlorine; each of which has peculiar virtues, the former in particular being a powerful oxidizing agent, the latter possessed of a great decomposing action over hydro-carbons, appears entitled to a high place among disinfectants. It can be cheaply procured by pouring oil of vitriol on a mixture of nitre and common salt, or by heating a mixture of nitric and muriatic acids.

One of the most rapid and effectual methods of disinfecting a large empty apartment such as an hospital ward, would be to place in one corner a vessel containing the materials for chlorine, such as oxide of manganese, common salt, and oil of vitriol; and in another corner a vessel containing nitric acid and a few fragments of copper, so as to evolve nitric acid, which would spread through the apartment and form nitrous acid there, oxidizing everything oxidable which it contained, whilst the chlorine specially attacked the hydrogenous compounds. The walls might then, if necesssry, be lime-washed, with a view alike to destroy any adhering organic matter which had resisted the action of the gases, and to neutralize any traces of free acid.

5. The last of the disinfectants proper to which I refer is the singular substance ozone, which has a special interest, as being in all probability the great natural disinfectant. Its nature is still matter of speculation. Schonbein, its discoverer, regards it as a peculiar oxide of hydrogen; Berzelius and Faraday represent it as simply oxygen in a peculiar (or allotropic) state of modification; it has been suggested that it is an oxide of nitrogen; and quite recently M. Fremy has affirmed it to be what he calls "electrized oxygen,"—*i. e.*, oxygen modified in properties by the action of electricity upon it; a view not materially differing from that of Berzelius and Faraday. There are difficulties in the way of all these views, into which it is not necessary to enter. All that concerns our present subject is that, by different processes a substance can be developed in the atmosphere which possesses remarkable disinfectant and oxidizing properties. The oldest known method of producing the so-called ozone, is the exposure of air to a stream of friction or high tension electricity. Its odor may always be recognized in the neighborhood of an electrical machine whilst at work. Another method is the galvanic decomposition of water, when the ozone accompanies the evolved oxygen. A third, and the most convenient method on the small scale, is the exposure of phosphorus in moist air. By these processes and by certain others, air is made to acquire a striking power of oxidizing, bleaching, deodorizing and disinfecting. We cannot doubt that every thunder-storm developes some ozone, and other processes also probably produce it. At all events the atmosphere frequently exhibits an oxidizing and bleaching power, at other times absent, which Schonbein, Faraday and others, attribute to the development of ozone within it.

No one who has experimented on ozone will doubt its potency. I refer to it here because there are so many reasons for believing that it is the agent which prevents the accumulation in the atmosphere of volatile organic bodies, by converting them into water, carbonic acid, nitric acid, and ammonia, that we cannot avoid looking hopefully to it as destined to prove our disinfectant *par excellence*. Certain as we are that for thousands of years miasmata, malaria, poisonous effluvia, and every gas, vapor, and volatile body developed at the surface of the earth, must have found their way into the atmosphere, and that nevertheless its purity is not sensibly affected, we must regard the constituent or condition of the air, which has secured its purity during centuries, as one demanding special study. Further this constant process of disinfection has not interfered with the respiration of animals, so that we may fairly regard ozone as a substance applicable as a disinfectant in places occupied by human beings or by the lower animals. It is true that the power of producing influenza or catarrh has been attributed to ozone in excess; on grounds, however, almost entirely speculative. This view may or may not be true; but of this I am quite certain, that the well-known impunity with which electricians expose themselves for hours together to the action on the atmosphere of large friction machines, which the dullest nostril can discover to be producing abundance of ozone, is enough to show that a large impregnation of the air with this substance, neither affects respiration nor produces catarrhal affections. We ought, therefore, I think, to give special attention to ozone. It is not likely that we shall be long without discovering new processes for its production. It will be specially valuable for what are the most important, and, at the same time, the most difficult occasions for disinfection—namely, where human beings cannot be removed from infected apartments. Examples of such cases are found in a large ship at all times, and still more when its crew and passengers are attacked by disease; in the wards of an hospital, from which the sick cannot be taken; and perhaps most strikingly in a large factory, where hundreds of persons assemble daily together, many of most uncleanly habits, and at epidemic seasons fresh from infected rooms, whilst the apartments contain valuable metallic machinery, and fragile silk, cotton, linen or wollen goods, which interpose an additional obstacle to the free employment of gaseous disinfectants. The condition of our ships as regards ventilation and wholesomeness is proverbial; and on inquiry of residents in Manchester and Glasgow, I find that where disinfection has been attempted in factories—which it rarely has—it has consisted in sending a man once a day through every room with a quantity of blazing pitch, which was supposed to fumigate into purity the atmosphere, whilst it set all the work-people coughing.

How difficult it is to prevent the spread of erysipelas, gangrene, fever, and the like in hospitals, every medical man knows too well. Ozone at least deserves a trial as a disinfectant in such cases.

*Antiseptics.*—The only antiseptics to which I shall refer are two. The first is sulphurous acid : it is a powerful antiseptic, for it resists thoroughly the decomposition or decay of organic matter. In reality, however, it as much resists the development as the decay of organic bodies, and thus it doubly prevents the evolution of organic poisons. Dr. Christison long ago pointed out how small a quantity of this acid is sufficient to destroy plants. In the wine countries it has been used from time immemorial to prevent the souring or acetification of the lighter wines, when kept in casks partially filled. Professor Graham, who strongly recommends it as a disinfectant, draws attention to the fact that at Manchester the offensive effluvia of the cochineal dye-vats, which resist the action of chlorine and nitric acid, are at once destroyed by sulphurous acid. My own attention was directed to it from the employment of it on a large scale by paper-makers and others to secure the preparation of pure gelatine, a substance peculiarly liable to enter into putrefaction. Sulphurous acid can be easily prepared by burning sulphur, or by heating oil of vitriol, along with charcoal or vegetable matter. Its corrosive action is very slight; its disinfecting action very powerful. The sulphite of soda is now prepared in quantity at different chemical works. The addition of a stronger acid sets free the sulphurous from its salts. As to its mode of action, if we concur with Liebig in believing that morbific matters resemble ferments in being active, only whilst undergoing a decomposition which is mainly determined by the oxygen of the air, we may suppose sulphurous acid to render the poisonous matter inert, by preventing its oxidation. This acid, moreover, is a powerful deoxidizing agent, and it may be by removing oxygen from organic poisons, that it renders them inert, by decomposing them.

Further, sulphurous acid can combine with certain elements of organic bodies, as we see in its temporary bleaching action on vegetable colors; and it may be thus that it neutralizes morbific matters. In one or other or all of those modes, this agent may act as a disinfectant; but at all events its action is very powerful, and it deserves much more attention than it has received.

The only other substance to which I shall at present refer, is pitch oil, one of the products of the distillation of tar. It is an antiseptic of the most powerful class, and very cheap, and if not used in excess it is applicable as a deodorizer, but its own strong tarry smell interferes with its extensive use.

